# Origin, Phylogeny, and Transmission of the Epidemic Clone ST208 of Carbapenem-Resistant Acinetobacter baumannii on a Global Scale

**DOI:** 10.1128/spectrum.02604-21

**Published:** 2022-05-31

**Authors:** Yue Gao, Henan Li, Hongbin Chen, Jiangang Zhang, Ruobing Wang, Zhiren Wang, Hui Wang

**Affiliations:** a Institute of Medical Technology, Peking University Health Science Center, Beijing, China; b Department of Clinical Laboratory, Peking University People's Hospital, Beijing, China; University of Pittsburgh School of Medicine

**Keywords:** *Acinetobacter baumannii*, ST208, whole genome sequencing (WGS), genome epidemiology

## Abstract

Carbapenem-resistant Acinetobacter baumannii (CRAB) is an opportunistic pathogen that has become a global threat. The dissemination of global clone 2 (GC2) CRAB has been well documented. Oxford sequence type (ST) 208 is one of the most prevalent lineages of A. baumannii GC2; however, its evolution and phylogeny are unclear. We collected 45 representative ST208 isolates from 14 cities in China between 1999 and 2018. Moreover, 411 ST208 genome sequences were downloaded from the GenBank database for comparison. The global ST208 phylogeny showed that ST208 might have originated from North America and subsequently evolved into two clades. Notably, the widespread OXA-23-producing ST208 A. baumannii was correlated with the transposon structure and dynamics of replicative transposition, and the Tn*2009* tandem structure of five copies of *bla*_OXA-23_ and potential circular intermediate of Tn*2009* were first detected. Furthermore, 15 Chinese ST208 isolates carried GR25 pABTJ1-like plasmids, which contained *bla*_OXA-23_ and have only been found in China in the last decade. In conclusion, our work suggests that replicative transposition contributes to the evolution and transmission of OXA-23-producing ST208 A. baumannii and highlights the new challenges posed by the epidemiological surveillance of globally distributed clonal groups via whole genome sequencing.

**IMPORTANCE** ST208 as one of the most prevalent lineages of CRAB has caused several difficult-to-treat infections and outbreaks around the world. However, few studies have focused on evaluating the genetic background differences of ST208 A. baumannii isolated from very distant geographic regions. A comprehensive genomic analysis of 456 clinical strains of ST208 A. baumannii from a wide temporal and geographic range was performed in this study. Moreover, the mechanisms leading to the horizontal transfer of *bla*_OXA-23_ in ST208 A. baumannii are poorly understood. We first describe experimental evidence of the potential circular intermediate of Tn*2009*, and the Tn*2009* tandem structure of five copies of *bla*_OXA-23_ was first detected. The interbacterial transfer of genetic elements carrying resistance to last-line antibiotic carbapenems highlights the essential need to enhance epidemiologic surveillance.

## INTRODUCTION

Acinetobacter baumannii has emerged as an opportunistic pathogen that has caused difficult-to-treat infections. Since the 1970s, it has spread rapidly across hospitals worldwide. Generally, A. baumannii is frequently involved in ventilator-associated pneumonia, wound infections, urinary tract infections, and bacteremia (1). These infections occur in hospitalized patients who have had significant contact with the health care system, especially in intensive care units (2). With a strong capacity for clonal transmission and the acquisition of antimicrobial resistance determinants, approximately 45% of global A. baumannii isolates are considered multidrug-resistant (MDR) (3). Outbreaks caused by MDR A. baumannii have been reported around the world. GC1 and GC2 are two major clones responsible for most of these outbreaks (4).

Clonal complex 208 (CC208), corresponding to GC2, is one of the largest clonal complexes of A. baumannii, which includes ST195 and ST208. CC208 often carries carbapenemase resistance genes such as *bla*_OXA-40-like_, *bla*_OXA-58-like_, and *bla*_OXA-23-like_ and the carbapenem resistance rate of CC208 is significantly higher than that of the non-CC208 group (1). ST208 A. baumannii was first detected in Australia in 1999 (5), and isolates were subsequently found in the United States and other European and Asian countries (6–9). In recent years, ST208 has become one of the predominant sequence types (STs) of carbapenem-non-susceptible isolates in China (10–12). Several outbreaks of MDR ST208 A. baumannii producing OXA-23-like carbapenemase have occurred (13–15), representing a serious public health threat. However, few studies have focused on evaluating the genetic background differences and worldwide dissemination of ST208 A. baumannii isolated from very distant geographic regions.

Recently, a study showed that neither the Oxford scheme nor the Pasteur scheme accurately reflects the relationships among A. baumannii isolates, especially for ST208 A. baumannii, and owing to the high levels of recombination events, ST208 could be divided into distinct lineages when employing cgMLST and cgSNP (16). However, the decrease in whole genome sequencing (WGS) costs would make this powerful genotyping strategy an ideal tool for analyzing A. baumannii with highly dynamic genomes (16). To increase our understanding of the genomic epidemiology, phylogenetic diversity, and evolution of ST208 A. baumannii, a comparative genomic analysis of 456 ST208 A. baumannii isolates recovered from 20 countries was performed.

## RESULTS

### Characteristics of ST208 A. baumannii in China.

According to our previous study (1999 to 2005) (17), the Chinese Antimicrobial Resistance Surveillance of Nosocomial Infections (CARES) (2007 to 2016) (18), and the Chinese Meropenem Surveillance Study (CMSS) (2010 to 2018) (19), we investigated the non-duplicate and clinically significant isolates from bloodstream infections, hospital-acquired pneumonia, and intra-abdominal infections from 1999 to 2018 in China prioritizing MDR A. baumannii and isolates mainly from blood, as well as from the respiratory tract. Then, 292 representative isolates consisting of 34 predominant clones in 13 cities of China and from different dates and sources were sequenced. In total, 45 ST208 A. baumannii isolates were selected in this study, consisting of two isolates from 2005, 10 isolates from CARES (2007 to 2016), and 33 isolates from CMSS (2010 to 2018), representing northern, eastern, central, and southern regions throughout China. Most of them were isolated from blood (25/45, 55.6%) and sputum (12/45, 26.7%).

### Phylogeny analysis of ST208 in a global context.

**FIG 1:**
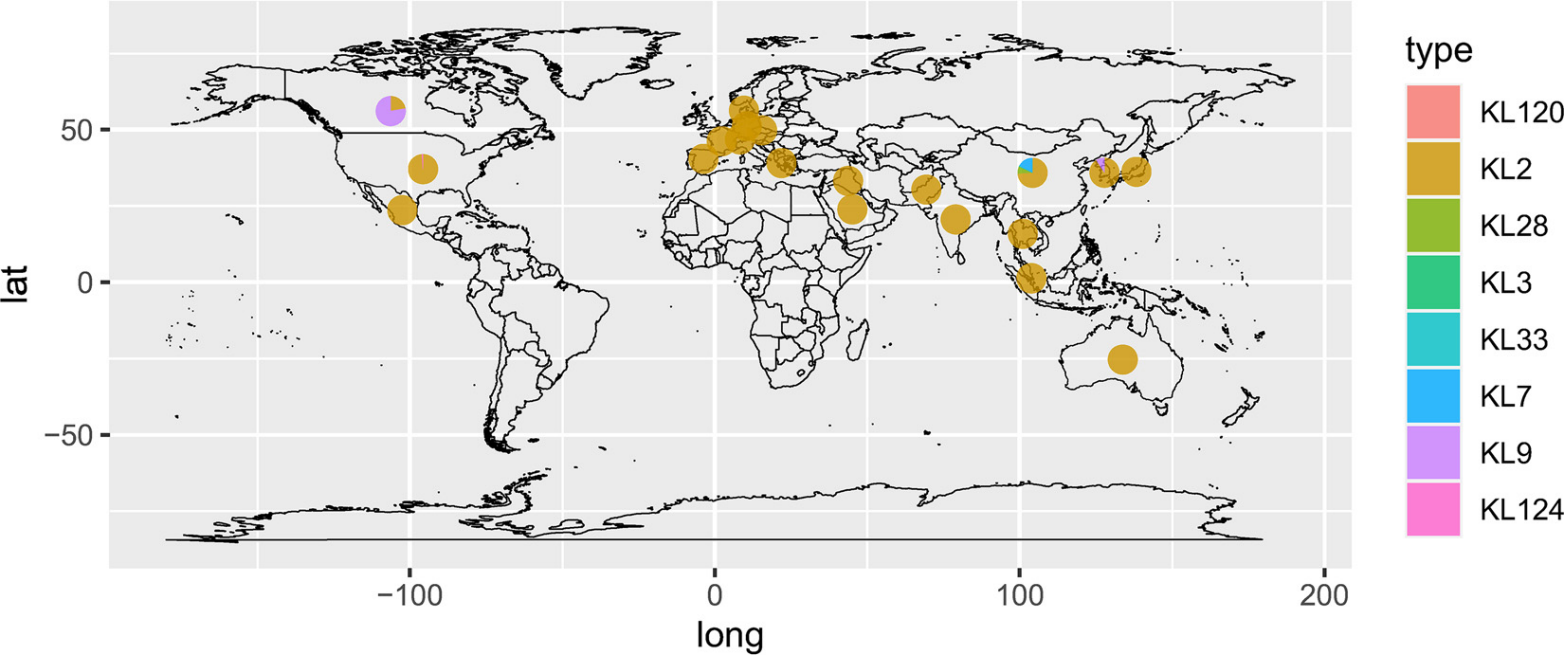
Geographic distribution of 456 ST208 Acinetobacter baumannii isolates involved in this study. Pie charts are colored based on the K type of the isolates.

For global phylogenetic analysis, 411 ST208 A. baumannii genome sequences with time data were downloaded from GenBank representing diverse geographical locations, including Australia (*n* = 2), Canada (*n* = 9), China (*n* = 177), Czech Republic (*n* = 1), Denmark (*n* = 2), France (*n* = 1), Germany (*n* = 3), Greece (*n* = 4), India (*n* = 3), Iraq (*n* = 1), Japan (*n* = 2), Mexico (*n* = 2), Pakistan (*n* = 7), Saudi Arabia (*n* = 2), Singapore (*n* = 1), South Korea (*n* = 11), Spain (*n* = 12), Switzerland (*n* = 3), Thailand (*n* = 7), the United States (*n* = 189), and unknown (*n* = 17) (Table S1). Capsular polysaccharides (CPSs) play an important role in enhancing resistance to disinfection and long periods of desiccation and contribute to the successful persistence of A. baumannii in the hospital environment (20). Capsule (K antigen) and/or O-antigen surface polysaccharides are major virulence determinants that protect A. baumannii from complement-mediated phagocytosis. The composition and structure of K antigens can vary considerably between different strains of the same species (21). The K types of 456 ST208 isolates were identified by adopting the Kaptive program in our study. KL2 was the most predominant K type, which is extensively found in North and Central America, Asia, Oceania, and Europe. As previously described, KL2 often occurs in antibiotic-resistant GC2 isolates from different sources and is geographically widely distributed (21, 22). Furthermore, KL7 and KL28 were only found in China, and KL9 was only found in some isolates from Canada and one isolate from South Korea (Fig. 1).

**FIG 2:**
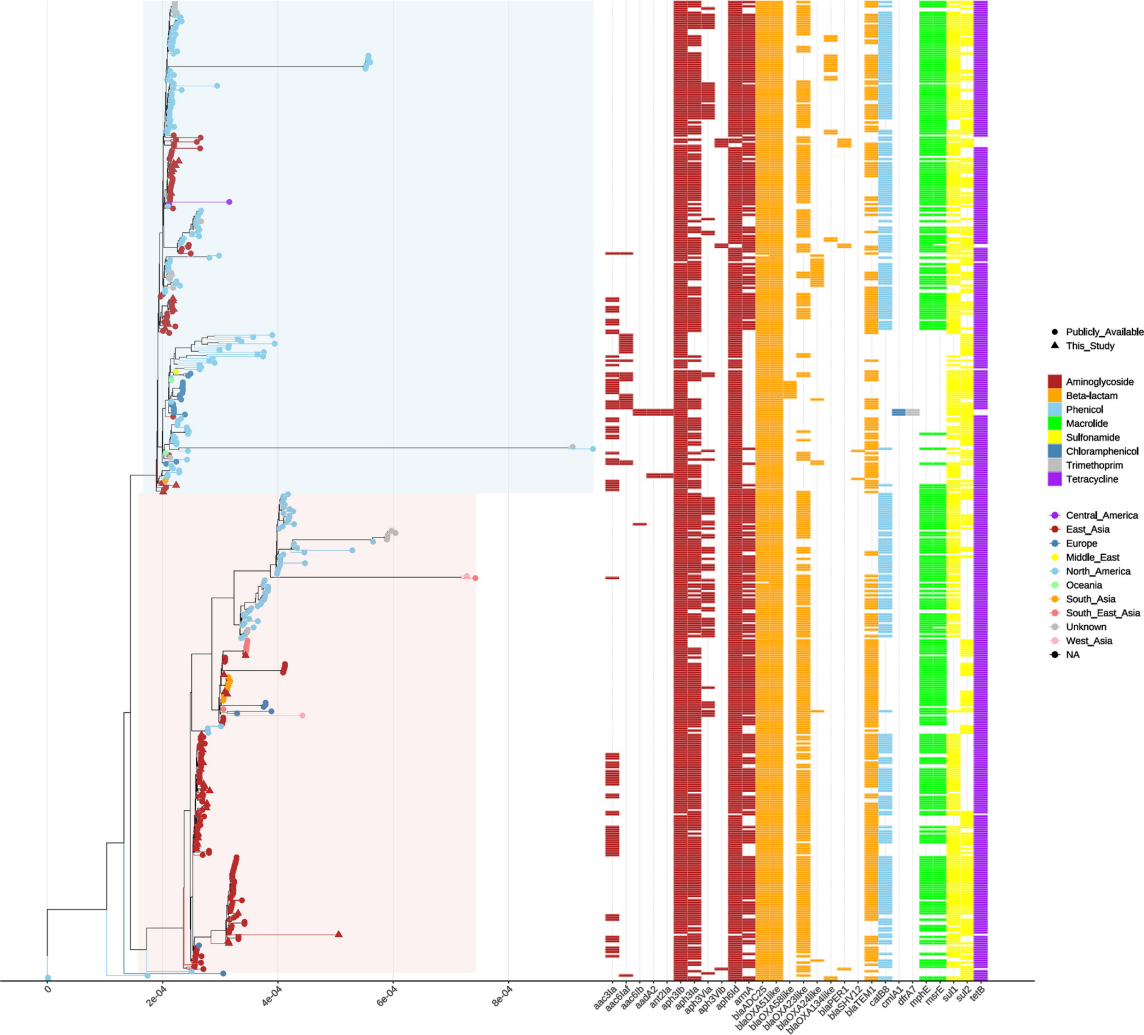
Recombination-filtered core genome phylogeny and the distribution of antimicrobial resistance genes in ST208 Acinetobacter baumannii. The highlighted boxes denote the substructure within ST208 (North America subclade and China subclade). The panel on the right indicates the presence or absence of the genes (colored or blank) carried per isolate. Identical colors for the strain name indicate that they were recovered from the same geographic location.

After excluding recombinant regions, a maximum-likelihood phylogenetic tree of all 456 ST208 A. baumannii genome sequences was then constructed (Fig. 2). The global ST208 phylogeny showed that ST208 might have originated from North America and subsequently evolved into two clades; most of the isolates from North America accumulated in one clade, and the other clade included isolates mainly from Asia, highlighting within-ST diversity. The close evolutionary relationships between isolates from geographically distant regions suggested the global dissemination of ST208 A. baumannii. Across the global core genome phylogenies, we found that Chinese isolates recapitulated a sizeable proportion of the diversity observed globally (Fig. 2), suggesting multiple introductions from and into China, likely with distinct phylogeographic origins.

### Variation in genes related to carbapenem resistance.

All 456 ST208 isolates carried some similar antimicrobial resistance (AMR) gene profiles, such as *aph(3′)-Ia* and *aph(6’)-Id* genes, encoding aminoglycoside resistance; the *tet(B)* gene, conferring resistance to tetracycline. However, the two subclades revealed different AMR gene profiles (Fig. 2). Compared with China subclade, there was considerable heterogeneity in the carriage of genes conferring resistance to aminoglycosides, beta-lactams, phenicols, macrolides, chloramphenicols, and trimethoprims within North America subclade. Some isolates from North America, Europe, Middle East, Oceania, and South Asia and East Asia in North America subclade lacked the genes *armA*, *bla*_OXA-23-like_, *catB8*, *mph(E)*, and *msr(E)*. Phylogeny across isolates suggested that the North America subclade and China subclade share a common ancestor, and then diverged from each other. However, they have been in joint circulation, possibly due to the clinical introduction of aminoglycosides, chloramphenicols, and trimethoprims, some isolates carried *aac(3′)-Ia*, *aac(6′)-Iaf*, *cmlA1*, and *dfrA7* (Fig. 2).

Although the short-read data produced by most currently available ST208 A. baumannii genomes are sufficient to identify antibiotic resistance genes or construct phylogenetic trees, they cannot resolve complex resistance regions (4), such as the OXA β-lactamases, which form the basis of transferable carbapenem resistance in A. baumannii (23). As numerous repeated elements in OXA resistance regions, with 387 short-read data and 69 long-read data of ST208 A. baumannii, we only detected the presence or absence of the following β-lactamase in this part. *bla*_OXA-66_ was the most common *bla*_OXA-51-like_ variant in ST208 A. baumannii (381/456, 84.9%) in our study. b*la*_OXA-23_ was present in 67.8% of ST208 isolates. The context of these complex resistance regions can be determined owing to optimal sequencing and assembly using long-read sequencing technologies such as PacBio or Oxford Nanopore Technology (ONT). In the following section of the accessory genome of ST208 A. baumannii, the upstream and downstream environments of *bla*_OXA-23_ in 69 long-read data sets were analyzed.

### Variation in the accessory genome of ST208.

The whole-genome repertoire of A. baumannii (the “pan genome”) is impressively large (24, 25), and as more genomes were collected, the core genome tended to become smaller and the accessory genome tended to become larger (24). The accessory genome represents an important component of the total A. baumannii genome and harbors elements that can be acquired by horizontal gene transfer. These include A. baumannii genomic resistance islands (AbGRIs), insertion sequence (IS) elements, plasmids, prophages, and transposons (25).

**FIG 3:**
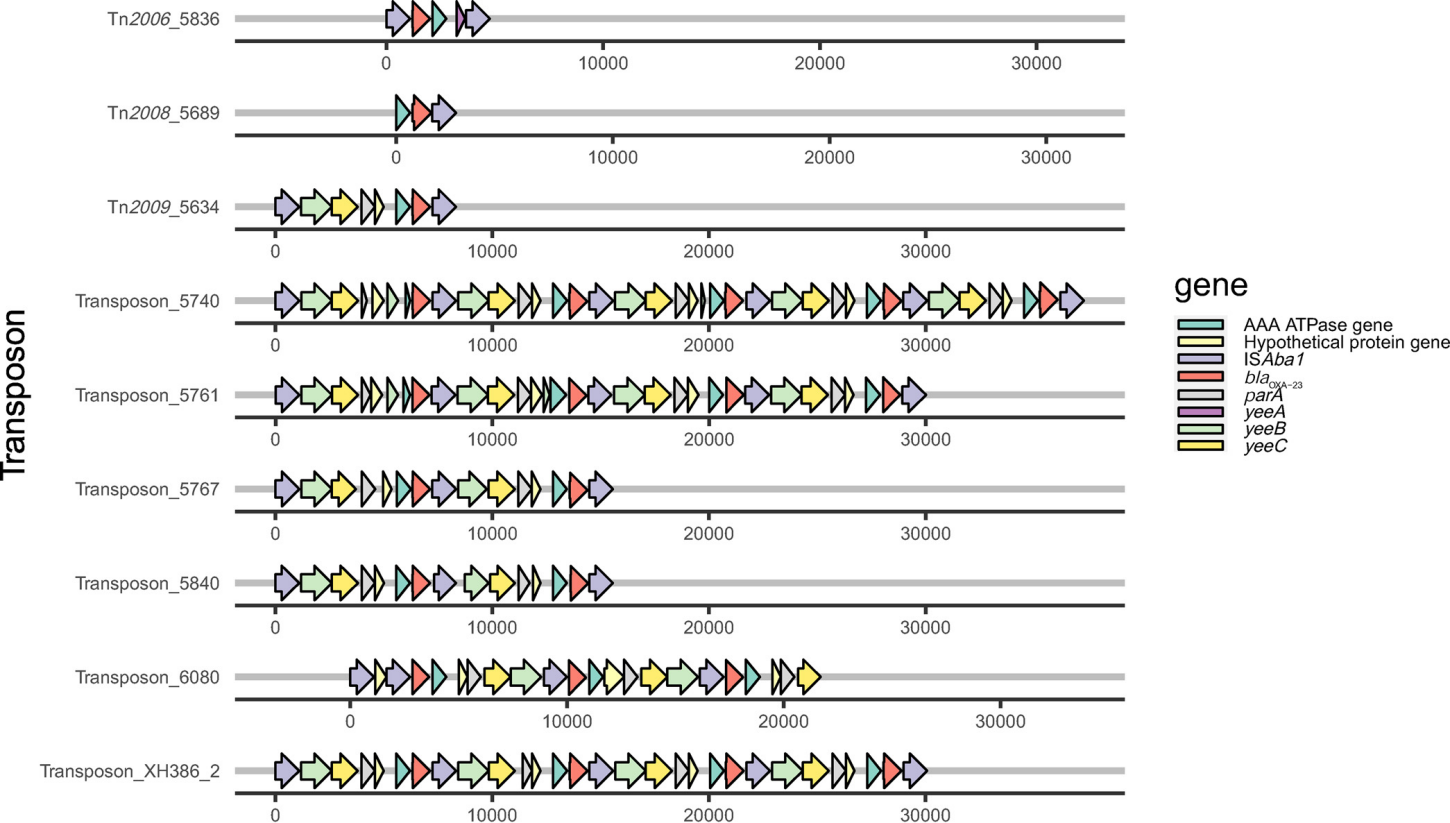
Genetic context of multiple copies of *bla*_OXA-23_ from six isolates. Isolates 5740, 5761, 5767, 5840, and XH386_2 showed several copies of the Tn*2009* unit present in tandem. Isolate 6080 consisted of two complete copies and an incomplete copy of Tn*2009*, which had three copies of *bla*_OXA-23_, and another IS*Aba1* was located upstream of this structure, forming a “duplicate” IS*Aba1*.

**FIG 4:**
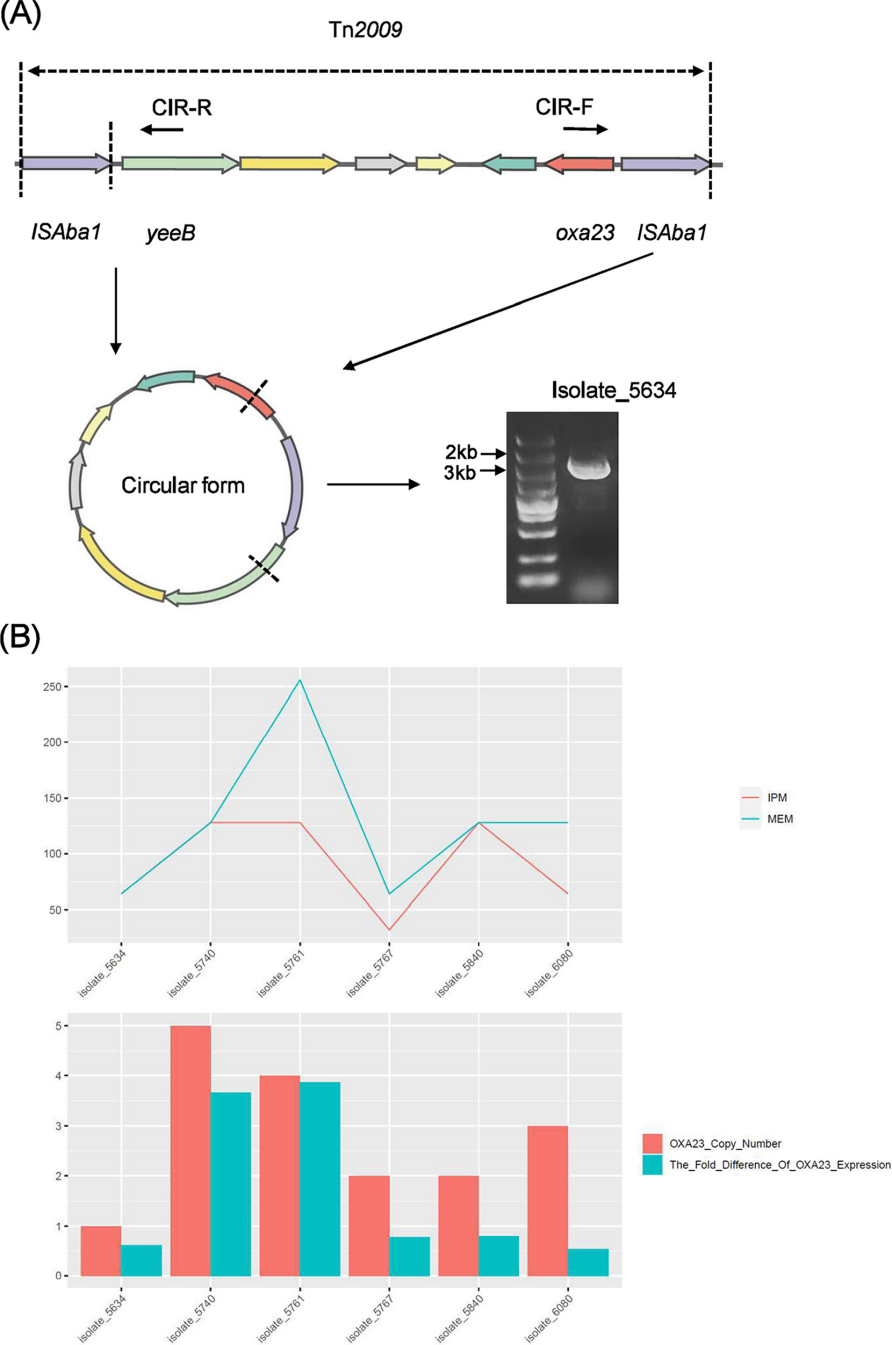
Formation of a potential circular intermediate by IS*Aba1.* (A) Genetic structure of transposon Tn*2009* (located in the chromosome of isolate 5634) and its potential circular intermediate. Gel electrophoresis of PCR amplicons corresponding to the circular intermediate of Tn*2009*, detectable in isolate 5634 using the reverse primers CIR-F and CIR-R. (B) MICs of imipenem (IPM) and meropenem (MEM) and the expression of *bla*_OXA-23_ in the six isolates. Isolate 6080 presented with lower expression of *bla*_OXA-23_ and MICs compared with those of the other five isolates.

We analyzed 69 ST208 PacBio sequencing data sets, including those from China (*n* = 49), Canada (*n* = 9), the United States (*n* = 5), Australia (*n* = 2), South Korea (*n* = 2), India (*n* = 1), and Mexico (*n* = 1). All strains showed distinct accessory genomes, and the accessory genomes of ST208 from China were more diverse than those from other places as they carried more transposons, prophage regions, and plasmids (Fig. S1). Valuable information about the origin, evolution, and spread of resistance in bacterial populations is often provided by the genetic environment of antibiotic resistance genes (4). *bla*_OXA-23_ is usually associated with plasmids or is integrated through transposons into the A. baumannii chromosome. Four transposons harboring *bla*_OXA-23_ have been reported, namely, Tn*2006*, Tn*2007*, Tn*2008*, and Tn*2009*. Tn*2007* possesses the IS*Aba4* promoter upstream of the *bla*_OXA-23_ gene and is thought to be immovable (not a transposon). Tn*2006*, Tn*2008*, and Tn*2009* share a common region, “OXA-23-ΔATPase.” They also contain IS*Aba1*. The two IS*Aba1* copies were inversely orientated in Tn*2006* compared with being the same direction in Tn*2009*. Tn*2006* and Tn*2008* are reported globally, whereas Tn*2009* has only been discovered in China (12). Of the 69 ST208 isolates, 40 carried at least one copy of *bla*_OXA-23_, and they were all located on transposons. Eleven isolates had multiple copies of *bla*_OXA-23_. In five isolates (5651, 5685, 5779, 5780, VB723), *bla*_OXA-23_ was located on Tn*2006* and scattered in different locations on the chromosomes; however, in six other isolates, *bla*_OXA-23_ copies clustered together to form a Tn*2009* tandem repeat structure (Fig. 3). Each of the *bla*_OXA-23_ genes was located in the same genetic context as the insertion sequence IS*Aba1* upstream and downstream and was bracketed by Tn*2009*. Two copies of IS*Aba1* were found to flank an internal segment that contains *the bla*_OXA-23_ gene, as well as genes encoding an AAA ATPase, hypothetical protein, ParA-like protein, YeeC-like protein, and DEAD helicase YeeB. Multiple copies of this unit were present in tandem. We hypothesized that *bla*_OXA-23_ translocation could be mediated via a circular intermediate with the assistance of the IS*Aba1* element. Reverse PCR was performed to detect the potential circular intermediate. PCR products with a size of ~2.3 kb were amplified from isolate 5634, which harbored only one copy of Tn*2009* (Fig. 4A). Sequence analysis showed that the PCR products consisted of partial sequences of *bla*_OXA-23_, *yeeB*, and one complete copy of IS*Aba1*, indicating the potential of Tn*2009* to form a circular intermediate via IS*Aba1*. After aligning this with the complete sequence of isolate 5634, we found that the IS*Aba1* element in the putative circular intermediate was derived from the downstream IS*Aba1* element, which differed from the upstream IS*Aba1* at the potential promoter region (upstream IS*Aba1*: ATATTT-IS*Aba1*, downstream IS*Aba1*: AAAGAG-IS*Aba1*). MICs of imipenem (IPM) and meropenem (MEM) correlated well with the expression of *bla*_OXA-23_ (Fig. 4B). However, isolate 6080, which had three copies of *bla*_OXA-23_, presented lower expression of *bla*_OXA-23_ and MICs compared with those of the other five isolates. It consisted of two complete copies and an incomplete copy of Tn*2009*, and another IS*Aba1* was located upstream of this structure, forming a “duplicate” IS*Aba1* (Fig. 3). Whether this structure affects the MICs of IPM and MEM and the expression of *bla*_OXA-23_ requires further investigation. A comparison of the gene-environment revealed that the tandem repeat structures were surrounded by a gene encoding the same hypothetical protein upstream and downstream. Previous studies have shown that IS*Aba1* is involved in the dissemination and amplification of *bla*_OXA-23_, although the Tn*2009* tandem structure of five copies of *bla*_OXA-23_ and the potential circular intermediate have not been previously reported.

AbaR-type islands are mainly found in the A. baumannii GC1 clone, and AbGRI1-type and AbGRI2-type islands occur mainly in A. baumannii GC2 isolates. As complex class III transposons, the AbGRI islands are located in the *comM* gene, with a set of *tniC–tniA–tniB–tniD–tniE* transposition genes in this location. AbGRI1 has a complex origin with various combinations of transposon Tn*6022* (*orf4-sup-uspA-orf-tniE-tniD-tniB-tniA-tniC*), a deletion derivative Tn*6022Δ*, a defective transposon Tn*6172*, *strA-strB* (streptomycin resistance), *tetA*(B) (tetracycline resistance), or *bla*_OXA-23_ from Tn*2006* (26). Interestingly, in our collection, except WM99c (isolated from 1999), all 68 genomes lacked *sul2* for sulfonamide resistance in AbGRI1 compared with the reference sequence A91 (GenBank accession number JN968483); however, *sul2* was still present in several ST208 A. baumannii genomes, suggesting that they might be found in other locations on their chromosomes.

### Comparison of ST208 plasmids.

We used 18 ST208 A. baumannii complete plasmid sequences deposited in NCBI as of December 15, 2020, and obtained 58 plasmids from our 45 isolates to increase the plasmid diversity included in our study. In total, our study comprised 76 plasmids of various sizes, ranging from 8,763 to 112,157 bp (Table S2). Moreover, our plasmid collection originated from 67 different isolates and five countries, each carrying up to three plasmids. In 2010, Bertini et al. made a classification system for A. baumannii plasmids based on the nucleotide identity of *rep* genes (27). In our study, the *rep* gene was compared with representatives of each group (GR) categorized by A. baumannii PCR-based replicon typing (AB-PBRT) using BLASTn to identify its closest matches. Among the 76 plasmids in our collection, 74 had an intact *rep* gene, including GR24 (*n* = 33), GR25 (*n* = 15), GR6 (*n* = 13), GR8 (*n* = 10), GR2 (*n* = 2), and GR26 (*n* = 1). Nevertheless, in plasmids p5741_1 and pORAB01-2, we could not find a Rep protein via BLAST searches or annotation (Table S2).

**FIG 5:**
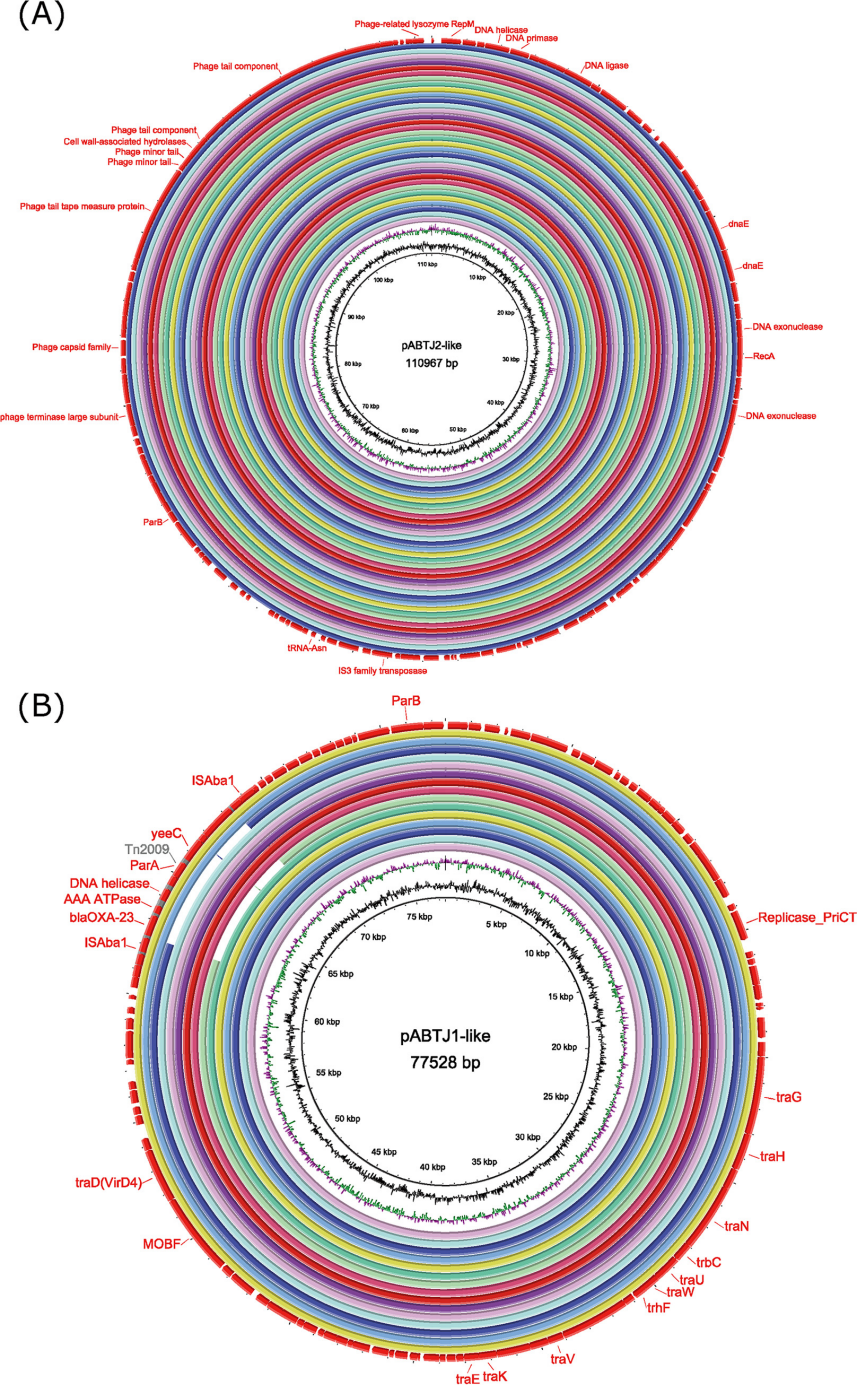
Comparative analyses of ST208 Acinetobacter baumannii plasmids. (A) Circular map of GR24 plasmids of ST208 A. baumannii. A cluster of genes encoding phage proteins is indicated in the outermost circle. Thirty-three plasmids with similar sequence organizations were aligned against pABTJ2 (GenBank accession number NC_020524). (B) Circular map of GR25 plasmids of A. baumannii ST208. Resistance determinants and type IV secretion systems are indicated in the outermost circle. Fifteen plasmids with similar sequence organizations were aligned against pABTJ1 (GenBank accession number CP003501).

The general structure of each plasmid group was very stable, even though some of the plasmids were isolated many years ago (28). As a representative plasmid of GR24, pABTJ2 was a 110,967 bp plasmid and contained many genes that normally form the chromosomes of bacteria or bacteriophages, such as *polA*, *dnaN*, *nrdA*, and *nrdB*, related to DNA metabolism and replication, which are speculated to improve replication function under stressful conditions. Several genes encoding phage proteins were scattered in the last 30 kb region of pABTJ2. These genes have been isolated and proposed to be phage remnants and encode proteins related to packing/morphogenesis and host lysis (29). In our study, 33 GR25 members were almost identical and shared very similar gene contents and organization. The oldest member of this group was pWM99c-2, isolated in 1999, and the most recent was p5846, isolated in 2018 (Fig. 5A). The presence of structurally nearly identical plasmids isolated from geographically widespread regions indicates the global dissemination of this type of stable plasmid in ST208 A. baumannii isolates.

Similarly, as a representative plasmid of GR25, the plasmid pABTJ1 was determined to be a 77 ,528 bp circular plasmid and harbored transposon Tn*2009*, flanked by two IS*Aba1* elements, transcribed in the same direction (30). pABTJ1 was isolated in 2012, and the sequences of 15 plasmids showed 99% identity to pABTJ1 (Fig. 5B); however, pABTJ1 was not the first GR25 plasmid to carry OXA-23 in China. According to our study, p5773 and p5729 were isolated as early as 2010. The *bla*_OXA-23_ gene located on a putatively conjugative plasmid might contribute to the dissemination of carbapenem resistance in this strain, and thus, it might have contributed to a wide host range in China over the last decade.

In general, the main types of plasmids in ST208 A. baumannii are GR24 and GR25. The GR24 pABTJ2-like plasmid has appeared in ST208 since 1999 and has been structurally stable, similar, and related to improving the replication functions of ST208 A. baumannii under stressful conditions. The GR25 pABTJ1-like plasmid was a newly discovered plasmid carrying *bla*_OXA-23_ in the last decade and has only been found in China to date, requiring surveillance of its further spread.

## DISCUSSION

Although cases of community-acquired infections have been reported, A. baumannii is undoubtedly related to hospital-acquired infections, mostly among immunocompromised patients of intensive care units. A. baumannii is primarily associated with ventilator-associated pneumonia, wound infections, urinary tract infections, and catheter-related bloodstream infections (31). It has been reported that ST208 and ST195 are the predominant epidemic types of MDR A. baumannii in China. According to our study, ST208 strains have been isolated from inpatients in several provinces of China since 2005. It is thought to be one of the most prevalent and widely distributed A. baumannii clones in China (15.4%, 45/292 isolates, collected from 14 cities between January 1999 and December 2018).

Owing to the high genome variation in A. baumannii, MLST schemes are probably not the best genotyping method (32). To assess the genetic relationship more accurately, we established a phylogenetic relationship among A. baumannii strains based on core genes. The close evolutionary relationships between isolates from geographically distant regions suggested the global dissemination of ST208 A. baumannii. The presence of some Chinese ST208 isolates in the North America subclade and vice versa further supports the spread of ST208 between continents. Phylogenetic analysis indicated that ST208 might have originated from North America and that it split into the North America subclade and China subclade. Possibly due to the clinical use of aminoglycosides, chloramphenicols, and trimethoprims, ST208 acquired extensive pan-genome diversity.

The OXA-type enzyme with potent carbapenemase activity was first described in 1985; it was a plasmid-encoded enzyme named ARI-1 and later named *bla*_OXA-23_. However, in recent decades, this enzyme type has typically contributed to carbapenem resistance in A. baumannii worldwide through horizontal gene transfer (33). Previous studies have shown that *bla*_OXA-23_ is the most common carbapenemase resistance gene in A. baumannii in China (34). *bla*_OXA-23_ was detectable in all 45 ST208 isolates in our study. Its expression is regulated by upstream promoters IS*Aba1* (35). The *bla*_OXA-23_ gene was found to be located on chromosomes and/or the plasmids and was associated with four different genetic structures, including Tn*2006*, Tn*2007*, Tn*2008*, and Tn*2009* (36). However, the horizontal transfer of these structures is poorly understood. In our study, multiple copies of *bla*_OXA-23_ clustered together to form a Tn*2009* tandem repeat structure, which was thought to form a circular intermediate to facilitate the dissemination of *bla*_OXA-23_. The co-existence of five copies of *bla*_OXA-23_ has not been previously reported. In addition, we found that 13 ST208 isolates from China carried the *bla*_OXA-23_ gene on GR25 pABTJ1-like plasmids and might be transferred horizontally to other species. Infection control measures and the rapid identification of bla*_OXA-23_* should be reinforced to reduce the spread of ST208 A. baumannii.

WGS remains the most powerful solution for global surveillance of genome epidemiology (37). Expanding the use of long-read sequencing will provide a better understanding of the mobile antibiotic resistance elements and their broader contexts. Completing genome and plasmid assemblies will facilitate the characterization of novel transposons and plasmids, also providing further knowledge regarding the widespread and significant horizontal gene transfer mechanisms. However, genomic sequence data from public databases without associated phenotypic data greatly limit the potential for the reuse of genomic sequence data to address further questions. Moreover, before WGS can be routinely employed for outbreak investigations, numerous simulations and optimizations should be conducted to make it more helpful and essential for surveying A. baumannii outbreaks.

## MATERIALS AND METHODS

### Bacterial isolates and antimicrobial susceptibility testing.

All 292 Chinese A. baumannii isolates were selected to cover wide temporal and geographic ranges and were characterized by both antibiotic resistance phenotypes and MLST according to the Oxford scheme (http://pubmlst.org/abaumannii/) (38). From this collection of isolates, 45 ST208 isolates were involved in this study. For global phylogenetic analysis, 411 total ST208 A. baumannii genome sequences were downloaded from GenBank, with other information, including time data, the isolation source, and geographical origins. MICs were determined using the broth microdilution method, and results were interpreted according to the standards of the Clinical and Laboratory Standards Institute (CLSI) 2021-M100 (http://www.clsi.org). Escherichia coli ATCC 25922 and Pseudomonas aeruginosa ATCC 27853 were used for quality control.

### Whole-genome sequencing, assembly, and annotation.

DNA from 45 ST208 isolates was isolated using a DNA purification kit (Qiagen). All isolates were subjected to WGS on the Pacific Biosciences (PacBio) Sequel platform and Illumina NextSeq 550 platform with 150-bp paired-end protocols. Libraries were prepared according to the manufacturer’s instructions. Hybrid assembly was performed using Unicycler (v0.4.6) (39) and annotated using Prokka (v1.12) (40).

### Phylogenetic analysis.

Conserved core genes among the ST208 isolates were analyzed using Roary (v3.11.2) (41) with a BLASTp percentage identity of 95 %. Possible recombination events were detected with ClonalFrameML (v1.2) (42) and Gubbins (v2.2.0) (43) and then removed. Maximum-likelihood phylogenetic trees were constructed using RAxML (v8.2.10) with a general time-reversible model and 1,000 bootstrap replicates (44). and the final tree was illustrated using the R ggtree package.

### Genome profiling.

Antimicrobial resistance genes were identified with ABRicate (v0.8.7) with the ResFinder database (45). The ISs were identified using the IS Finder database (http://www-is.biotoul.fr/) (46). The locus encoding proteins responsible for biosynthesis and export (K locus) and the locus for outer core biosynthesis (OC locus) of CPS were identified with Kaptive (v0.7.0) (47). Phage-associated regions were identified using PHASTER (http://phaster.ca/) (48). We obtained the complete plasmid sequences of the 45 isolates using the PacBio RSII and Illumina NextSeq platforms. Relevant plasmids were searched in the NCBI nucleotide and plasmid databases. All complete plasmids of ST208 A. baumannii available in the RefSeq and GenBank databases before December 15, 2020, were downloaded. Functional annotation was performed using RAST v2.0 (https://rast.nmpdr.org/rast.cgi) (49). Plasmid sequences were compared using BLASTn and illustrated using BLAST Ring Image Generator (BRIG) (50).

### Detection of potential circular intermediates of Tn*2009*.

Reverse PCR (CIR-F, 5′-TATTTGCGCGGCTTAGAGCA-3′, and CIR-R, 5′-ATCGTTAGTGTTCCTGGCGG-3′; annealing temperature 56°C; amplicon size 2318 bp) was performed to investigate the potential of the IS*Aba1* segment to circularize. The PCR product was then sequenced by Sanger sequencing.

### Determination of *bla*_OXA-23_ expression.

The expression levels of *bla*_OXA-23_ were analyzed by reverse transcription quantitative PCR (RT-qPCR) with specific primers (OXA23-F, 5′-TGACCTTTTCTCGCCCTTCC-3′, OXA23-R, 5′-TGCTCTAAGCCGCGCAAATA-3′, rpoB-F, 5′-CATTGGTGCCGCATAAGTCG-3′, and rpoB-R 5′-CTCCAAGCCGCATTTCGTTC-3′). Briefly, bacteria were grown in LB broth until mid-log phase. DNase-treated RNA templates were prepared using the Qiagen RNeasy minikit. cDNA was generated from total RNA using random primer hexamers. Dilutions of cDNA were used to quantify the level of *bla*_OXA-23_ by RT-qPCR, performed on an Applied Biosystems (ABI) 7500 real-time PCR system with a SYBR green PCR master mix (TaKaRa). The housekeeping gene *rpoB* was used for the calculation of relative expression.

### Data availability.

All 45 complete genome sequences were deposited in the National Center for Biotechnology Information (NCBI) database. 40 newly uploaded genomes are available with the BioProject accession number PRJNA789460 and for 5 previously uploaded genomes can be found at GenBank under the accession numbers CP059354 (5662), CP082225 (p5662), CP059353 (5665), CP082892 (p5665_1), CP082891 (p5665_2), CP082893 (p5665_3), CP059352 (5678), CP082888 (p5678_1), CP082889 (p5678_2), CP082890 (p5678_3), CP079942 (5780), CP059359 (5836), CP082221 (p5836).
