# Supplementary material for: Origin, Phylogeny, and Transmission of the Epidemic Clone ST208 of Carbapenem-Resistant Acinetobacter baumannii on a Global Scale
Source: Microbiol Spectr. 2022 May 31;10(3):e02604-21. doi: 10.1128/spectrum.02604-21 (PMC9241911; doi:10.1128/spectrum.02604-21)
Supplement: SUPPLEMENTAL FILE 1 — Supplemental material. Download spectrum.02604-21-s0001.pdf, PDF file, 0.7 MB [file spectrum.02604-21-s0001.pdf]

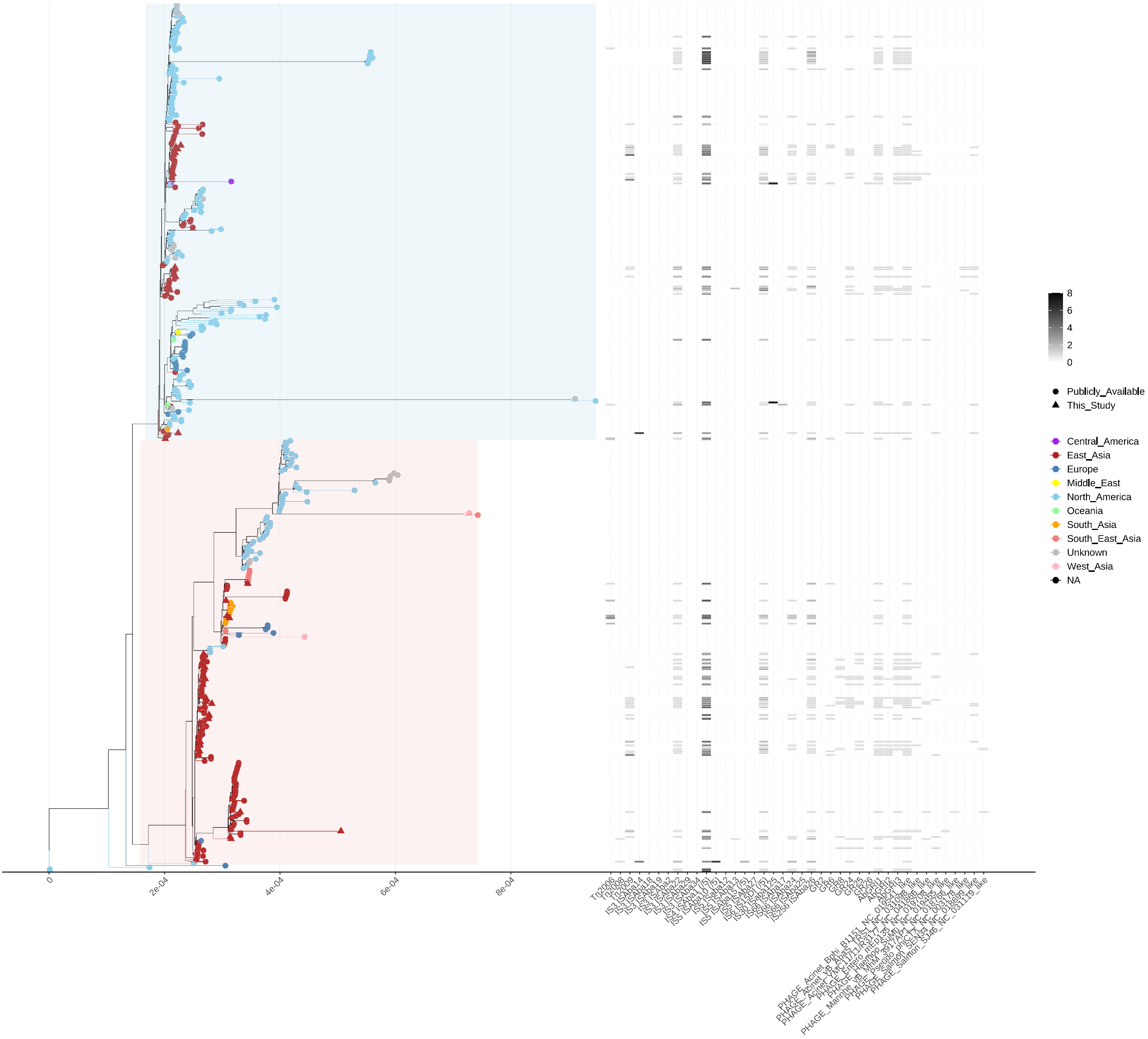

Fig. S1. The accessory genome of 69 ST208. Recombination-filtered core genome phylogeny of ST208 *A. baumannii.* The copy number of accessory genome elements in an isolate is denoted by the heatmap. Included elements are: *A. baumannii* genomic resistance islands (AbGRIs), insertion sequence (IS) elements, plasmids, prophages, and transposons.

Table S1. Sequences of 456 ST208 *A. baumannii* isolates used for phylogenetic analysis in this study

Strain	Isolation source	Country	Collection date	Accession number	K Type
80	Cerebrospinal Fluid	Spain	07/02/2008	MDTR01	KL2
314	Cerebrospinal Fluid	Spain	07/07/2008	MDTT01	KL2
428	Cerebrospinal Fluid	Spain	07/11/2008	MDTS01	KL2
5388	Blood	China	12/26/2016	This Study	KL2
5626	Sputum	China	07/01/2005	This Study	KL2
5634	Sputum	China	03/01/2013	This Study	KL2
5651	Sputum	China	07/01/2005	This Study	KL3
5653	Sputum	China	05/09/2013	This Study	KL2
5656	Blood	China	07/17/2014	This Study	KL2
5662	Blood	China	04/07/2014	This Study	KL2
5663	Blood	China	06/16/2014	This Study	KL2
5664	Blood	China	05/05/2014	This Study	KL2
5665	Cerebrospinal Fluid	China	03/18/2016	This Study	KL2
5666	Blood	China	03/17/2016	This Study	KL2
5669	Blood	China	05/02/2013	This Study	KL2
5670	Blood	China	06/01/2013	This Study	KL2
5671	Blood	China	03/08/2013	This Study	KL2
5672	Blood	China	06/01/2013	This Study	KL33
5678	Blood	China	05/05/2014	This Study	KL2
5679	Blood	China	03/17/2014	This Study	KL2
5683	Blood	China	01/20/2016	This Study	KL2
5685	Blood	China	07/13/2016	This Study	KL2
5689	Blood	China	05/05/2016	This Study	KL120
5729	Secretion	China	11/03/2010	This Study	KL2
5732	Sputum	China	12/03/2010	This Study	KL2
5734	Blood	China	06/29/2010	This Study	KL2
5736	Sputum	China	12/14/2010	This Study	KL2
5740	Sputum	China	11/16/2010	This Study	KL2
5741	Sputum	China	12/03/2010	This Study	KL2
5745	Abdominal Fluid	China	10/05/2011	This Study	KL2
5759	Balf	China	04/25/2012	This Study	KL2
5760	Sputum	China	05/29/2012	This Study	KL2
5761	Blood	China	02/01/2012	This Study	KL2
5765	Blood	China	11/15/2010	This Study	KL2
5767	Blood	China	07/10/2012	This Study	KL2
5768	Blood	China	04/10/2012	This Study	KL2
5769	Bronchial	China	08/01/2012	This Study	KL2
5771	Balf	China	05/07/2012	This Study	KL2
5773	Secretion	China	03/03/2012	This Study	KL2
5779	Sputum	China	12/13/2018	This Study	KL2
5780	Sputum	China	12/13/2018	This Study	KL2
5836	Blood	China	02/04/2018	This Study	KL2
5839	Blood	China	05/11/2018	This Study	KL7
5840	Blood	China	11/13/2018	This Study	KL2
5846	Blood	China	09/06/2018	This Study	KL2
5847	Sputum	China	07/18/2016	This Study	KL2
5955	Tracheal Aspirate	China	10/25/2018	This Study	KL2
6080	Blood	China	10/30/2018	This Study	KL7
7847	Blood	Mexico	04/28/2008	CP023031	KL2
11126	Sputum	USA	07/01/2012	JFWS01	KL2
20881	Sputum	China	06/22/2017	VMID01	KL2
20883	Sputum	China	07/24/2017	VMIF01	KL2
48055	Tracheal Secretion	Denmark	07/01/2010	AOSP01	KL2
53264	Tracheal Secretion	Denmark	07/01/2010	ALPW01	KL2
70136	Sputum	USA	07/01/2011	JFYK01	KL2
145660	Sputum	USA	07/01/2011	JFXU01	KL2
323408	Sputum	USA	07/01/2012	JFCZ01	KL2
339786	Sputum	USA	07/01/2012	JFXO01	KL2
730795	Sputum	USA	07/01/2012	JFYB01	KL2
825610	Perirectal	USA	07/01/2012	JFEZ01	KL2
1051176	Perirectal	USA	07/01/2008	JEZB01	KL2
1146103	Perirectal	USA	07/01/2012	JMNS01	KL2
1277411	Perirectal	USA	07/01/2012	JEXL01	KL2
1291820	Perirectal	USA	07/01/2012	JEXK01	KL2
1440750	Perirectal	USA	07/01/2012	JEXQ01	KL2

1488685	Sputum	USA	07/01/2012	JFCT01	KL2
1539026	Sputum	USA	07/01/2012	JMNH01	KL2
1552389	Sputum	USA	07/01/2012	JFCS01	KL2
1552865	Perirectal	USA	07/01/2012	JEXY01	KL2
09A16CRGN0014	Unknown	Canada	07/01/2016	CP034242	KL2
09A16CRGN003B	Unknown	Canada	07/01/2016	CP034243	KL2
11A1213CRGN008	Unknown	Canada	07/01/2012	CP035186	KL9
11A1213CRGN055	Unknown	Canada	07/01/2012	CP035185	KL9
11A1213CRGN064	Unknown	Canada	07/01/2012	CP043419	KL9
11A1314CRGN088	Unknown	Canada	07/01/2013	CP035184	KL9
11A1314CRGN089	Unknown	Canada	07/01/2013	CP043418	KL9
11A14CRGN003	Unknown	Canada	07/01/2014	CP035183	KL9
1276470_132	Sputum	USA	07/01/2012	JMOK01	KL2
2005JSAB1	Sputum	China	07/01/2005	JSCO01	KL2
2005LNAB4	Sputum	China	07/01/2005	JSCU01	KL2
2011BJAB2	Sputum	China	07/01/2011	JRHW01	KL2
2011BJAB5	Blood	China	07/01/2011	JSCY01	KL2
2011BJAB6	Abdominal Fluid	China	07/01/2011	JSDM01	KL2
2011BJAB8	Sputum	China	07/01/2011	JSCZ01	KL2
2011HNAB1	Sputum	China	07/01/2011	JSDF01	KL2
2011SDAB2	Abdominal Fluid	China	07/01/2011	JSCR01	KL2
2011ZJAB3	Blood	China	07/01/2011	JSDK01	KL2
2015ZJAB15	Sputum	China	07/01/2014	WQMD01	KL2
2015ZJAB16	Sputum	China	07/01/2014	WQME01	KL2
2015ZJAB7	Drain Fluid	China	01/04/2013	LQCQ01	KL7
2015ZJAB8	Drain Fluid	China	01/14/2014	LQCR01	KL7
AB_025	Environmental	Pakistan	01/15/2016	RIAI01	KL2
AB_029	Environmental	Pakistan	01/15/2016	RIAG01	KL2
AB_147	Environmental	Pakistan	01/15/2016	RHZG01	KL2
AB_152	Environmental	Pakistan	01/15/2016	RHZE01	KL2
AB_368	Environmental	Pakistan	12/15/2016	RHYD01	KL2
AB_369	Environmental	Pakistan	12/15/2016	RHYC01	KL2
AB_HZ_B09	Blood	China	03/15/2014	PRJI01	KL2
AB_HZ_B13	Blood	China	01/19/2014	PRJE01	KL2
AB_HZ_B29	Blood	China	06/20/2014	PRIT01	KL2
AB_HZ_B40	Blood	China	01/09/2015	PRIH01	KL2
AB_HZ_B43	Blood	China	01/28/2015	PRIE01	KL2
AB_HZ_B45	Blood	China	02/15/2015	PRJM01	KL2
AB_HZ_S06	Sputum	China	09/30/2014	PRGV01	KL2
AB_HZ_S13	Sputum	China	11/27/2013	PRGI01	KL2
AB_HZ_S17	Sputum	China	01/18/2014	PRHE01	KL2
AB_HZ_S29	Sputum	China	06/17/2014	PRFX01	KL2
AB_HZ_S40	Sputum	China	01/08/2015	PRFL01	KL2
AB_HZ_S43	Sputum	China	01/28/2015	PRFI01	KL2
AB_HZ_S56	Sputum	China	01/17/2014	PRHV01	KL7
AB_HZ_S90	Sputum	China	05/03/2013	PRGR01	KL2
AB_HZ_S91	Sputum	China	05/05/2013	PRGQ01	KL2
AB_US_1	Environmental	USA	01/15/2016	RHXP01	KL2
AB_US_10	Environmental	USA	02/15/2016	RHXQ01	KL2
AB_US_2	Environmental	USA	01/15/2016	RHXO01	KL2
AB_US_6	Environmental	USA	01/15/2016	RHXN01	KL2
AB10	Unknown	China	07/01/2000	QPHT01	KL2
AB100	Sputum	China	11/21/2015	NKJY01	KL7
AB101	Bile	China	11/21/2015	NKJX01	KL2
AB108	Sputum	China	11/24/2015	NKJW01	KL2
AB112	Sputum	China	11/26/2015	NKJV01	KL7
AB117	Sputum	China	04/15/2015	VAQJ01	KL7
AB118	Pus	China	11/27/2015	NKJU01	KL2
AB119	Sputum	China	04/18/2015	VAQI01	KL7
AB120	Catheter Liquid	China	04/29/2015	VAQH01	KL2
AB133	Sputum	China	07/02/2015	VAQE01	KL7
AB134	Sputum	China	12/03/2015	NKJT01	KL28
AB139	Sputum	China	07/28/2015	VAQB01	KL2
AB143	Blood	China	08/12/2015	VAPZ01	KL7
AB147	Sputum	China	12/10/2015	NKJS01	KL2
AB156	Sputum	China	12/14/2015	NKJR01	KL28
AB159	Blood	China	12/23/2015	VARG01	KL7

Ab16	Sputum	China	07/30/2013	VAMU01	KL2
AB161	Sputum	China	12/14/2015	NKJQ01	KL28
AB162	Sputum	China	12/15/2015	NKJP01	KL7
AB165	Arteriovenous Catheter	China	12/18/2015	NKJO01	KL28
AB166	Sputum	China	12/19/2015	NKJN01	KL2
AB172	Sputum	China	12/25/2015	NKJM01	KL28
AB173	Sputum	China	12/26/2015	NKJL01	KL2
AB174	Sputum	China	12/27/2015	NKJK01	KL2
Ab191	Sputum	China	09/15/2018	VAMH01	KL2
AB1H8	Sputum	China	07/01/2005	ANNC01	KL2
Ab21	Sputum	China	08/19/2013	VAMX01	KL2
AB217	Wound Swab	Saudi Arabia	07/01/2012	LXJV01	KL2
Ab23	Sputum	China	08/21/2013	VALN01	KL2
AB250	Sputum	Saudi Arabia	07/01/2012	LXJW01	KL2
AB46	Sputum	China	11/02/2015	NKKN01	KL2
Ab49_GEIH_2010	Unknown	Spain	07/01/2010	MSMM01	KL2
AB52	Sputum	China	11/02/2015	NKKM01	KL7
AB54	Sputum	China	11/04/2015	NKKL01	KL7
AB56	Vaginal Secretion	China	11/04/2015	NKKK01	KL2
AB7	Unknown	China	07/01/2011	QPHV01	KL2
AB72	Sputum	China	11/10/2015	NKKJ01	KL2
AB73	Sputum	China	11/11/2015	NKKI01	KL7
AB74	Arteriovenous Catheter	China	11/12/2015	NKKH01	KL28
AB75	Sputum	China	11/14/2015	NKKG01	KL2
Ab75	Sputum	China	09/09/2014	VAOH01	KL2
AB79	Eye Secretion	China	11/16/2015	NKKF01	KL28
AB8	Unknown	China	07/01/2003	QPHU01	KL2
AB81	Sputum	China	11/16/2015	NKKE01	KL28
AB88	Sputum	China	11/16/2015	NKKD01	KL28
AB89	Sputum	China	11/16/2015	NKKC01	KL28
AB91	Sputum	China	11/17/2015	NKKB01	KL2
Ab91	Sputum	China	01/30/2015	VANZ01	KL2
AB96	Umbilical Cord Secretions	China	11/18/2015	NKKA01	KL28
AB99	Umbilical Cord Secretions	China	11/21/2015	NKJZ01	KL28
ABAY04001	Blood	South Korea	07/01/2004	RPSL01	KL2
ABAY11005	Blood	South Korea	06/14/2011	QHFU01	KL2
ABAY12014	Blood	South Korea	10/24/2012	QHEW01	KL2
ABAY12015	Blood	South Korea	10/24/2012	QHEV01	KL2
ABAY12016	Blood	South Korea	11/04/2012	QHEU01	KL2
ABAY13005	Blood	South Korea	03/14/2013	QHEM01	KL2
ABBL008	Blood	USA	08/11/2005	LLCO01	KL2
ABBL060	Blood	USA	09/15/2008	LLFF01	KL2
ABBL109	Blood	USA	09/06/2010	LLHH01	KL2
ABBL115	Blood	USA	02/21/2011	LLHM01	KL2
ABBL116	Blood	USA	02/26/2011	LLHN01	KL2
ABBL121	Blood	USA	07/25/2011	LLHR01	KL2
ABBL123	Blood	USA	08/23/2011	LLHT01	KL2
ABBL124	Blood	USA	09/03/2011	LLHU01	KL2
ABBL137	Blood	USA	05/14/2012	LLIG01	KL2
ABBL141	Blood	USA	06/12/2012	LLIJ01	KL2
ABCRPUTH04	Patient	Thailand	09/07/2016	NQFA01	KL2
ABCRPUTH06	Patient	Thailand	09/14/2016	NQFC01	KL2
ABCRPUTH08	Patient	Thailand	12/09/2016	NQFE01	KL2
ABCRPUTH12	Patient	Thailand	07/28/2016	NQFI01	KL2
ABCRPUTH14	Patient	Thailand	11/09/2016	NQFK01	KL2
AbMDR_GLH1	Tracheobronchial Aspirate	Spain	01/27/2011	LIWE01	KL2
AbMDR_GLH10	Tracheobronchial Aspirate	Spain	01/17/2011	LJAH01	KL2
AbMDR_GLH11	Tracheobronchial Aspirate	Spain	01/24/2011	LJAI01	KL2
AbMDR_GLH2	Tracheobronchial Aspirate	Spain	02/03/2011	LIZZ01	KL2
AbMDR_GLH3	Tracheobronchial Aspirate	Spain	02/04/2011	LJAA01	KL2
AbMDR_GLH4	Tracheobronchial Aspirate	Spain	05/03/2011	LJAB01	KL2
AbMDR_GLH8	Tracheobronchial Aspirate	Spain	03/15/2011	LJAF01	KL2
AbMDR_GLH9	Tracheobronchial Aspirate	Spain	04/25/2011	LJAG01	KL2
ABUH337	Sputum	USA	01/17/2009	MWVN01	KL2
ABUH339	Sputum	USA	01/28/2009	MWVO01	KL2
ABUH341	Wound Abscess	USA	01/31/2009	MWVP01	KL2
ABUH343	Sputum	USA	02/02/2009	MWVQ01	KL2

ABUH345	Sputum	USA	02/18/2009	NCZL01	KL2
ABUH347	Urine	USA	02/22/2009	NCZM01	KL2
ABUH353	MISC	USA	03/11/2009	NCZN01	KL2
ABUH356	Blood	USA	03/18/2009	NCZO01	KL2
ABUH367	Wound Abscess	USA	04/29/2009	NCZS01	KL2
ABUH371	Sputum	USA	04/30/2009	NCZT01	KL2
ABUH374	Sputum	USA	05/19/2009	NCZU01	KL2
ABUH375	Urine	USA	06/07/2009	NCYJ01	KL2
ABUH379	Wound Abscess	USA	06/20/2009	NCYK01	KL2
ABUH383	MISC	USA	07/19/2009	NCYL01	KL2
ABUH388	Biopsy	USA	07/30/2009	NCYM01	KL2
ABUH394	Sputum	USA	08/04/2009	NCYN01	KL2
ABUH413	Urine	USA	08/28/2009	NCYR01	KL2
ABUH424	Sputum	USA	09/20/2009	NCYT01	KL2
ABUH432	Wound Abscess	USA	10/22/2009	MSNC01	KL2
ABUH434	Sputum	USA	10/22/2009	NCYX01	KL2
ABUH435	Sputum	USA	10/22/2009	MSND01	KL2
ABUH437	Sputum	USA	10/27/2009	NCYY01	KL2
ABUH438	Sputum	USA	10/29/2009	MSNE01	KL2
ABUH441	Wound Abscess	USA	11/06/2009	MSNF01	KL2
ABUH449	Sputum	USA	11/23/2009	NCZB01	KL2
ABUH453	Sputum	USA	11/30/2009	MSPB01	KL2
ABUH464	Blood	USA	12/28/2009	NCZF01	KL2
ABUH475	Sputum	USA	01/28/2010	NCZH01	KL2
ABUH496	Sputum	USA	04/11/2010	NCZK01	KL2
ABUH499	Sputum	USA	01/07/2011	NCXT01	KL2
ABUH504	Sputum	USA	01/17/2011	MSPK01	KL2
ABUH508	Urine	USA	02/24/2011	NCXW01	KL2
ABUH519	Sputum	USA	06/02/2011	MSNX01	KL2
ABUH522	Sputum	USA	07/06/2011	MSNY01	KL2
ABUH525	Sputum	USA	06/27/2011	MSNZ01	KL2
ABUH529	Tracheal Aspirate	USA	07/17/2011	NCYA01	KL2
ABUH532	Sputum	USA	07/30/2011	NCYB01	KL2
ABUH533	Urine	USA	08/28/2011	NCYC01	KL2
ABUH534	Tracheal Aspirate	USA	08/10/2011	MSOA01	KL2
ABUH542	Sputum	USA	09/12/2011	NCYD01	KL2
ABUH555	Sputum	USA	12/08/2011	MSOG01	KL2
ABUH582	Sputum	USA	04/05/2012	MSON01	KL2
ABUH586	Sputum	USA	04/13/2012	NDAR01	KL2
ABUH592	Sputum	USA	06/09/2012	NDAT01	KL2
ABUH594	Sputum	USA	06/17/2012	MSOR01	KL2
ABUH596	Sputum	USA	06/25/2012	NDAU01	KL2
ABUH604	Tracheal Aspirate	USA	07/28/2012	NDAV01	KL2
ABUH618	Wound Abscess	USA	10/15/2012	MSOX01	KL2
ABUH625	Wound Abscess	USA	11/24/2012	NDBA01	KL2
ABUH627	Stool	USA	12/31/2012	MSOZ01	KL2
ABUH632	Wound Abscess	USA	01/21/2013	NCZX01	KL124
ABUH634	Urine	USA	02/22/2013	NCZY01	KL2
ABUH657	Urine	USA	06/30/2013	MSNO01	KL124
ABUH678	Wound Abscess	USA	09/17/2013	NDAI01	KL2
ABUH687	Sputum	USA	12/03/2013	MSNU01	KL2
ABUH690	Blood	USA	12/14/2013	MSNV01	KL2
ABUH691	Tracheal Aspirate	USA	12/14/2013	NDAL01	KL2
ABUH692	Sputum	USA	12/09/2013	NDAM01	KL2
ABUH722	Unknown	Unknown	07/15/2014	LZTY01	KL2
ABUH733	Unknown	Unknown	09/14/2014	LZWK01	KL2
ABUH735	Unknown	Unknown	09/23/2014	LZVQ01	KL2
ABUH743	Unknown	Unknown	11/22/2014	LZVR01	KL124
ABUH768	Unknown	Unknown	04/15/2005	LZXC01	KL124
AC002	Rectal Swab	Switzerland	07/01/2010	LNUZ01	KL2
AC002_1_R4	Rectal Swab	Switzerland	07/01/2015	LNVE01	KL2
AC002_1_R4_S1	Rectal Swab	Switzerland	07/01/2015	LNVD01	KL2
Aci00844	Rectal Swab	Germany	05/12/2015	VAFW01	KL2
Aci00862	Tracheal Secretion	Germany	04/29/2013	VAGG01	KL2
Aci00863	Tracheal Secretion	Germany	09/02/2013	VAGH01	KL2
AF-673	Sputum	USA	03/20/2008	CP018256	KL2
ARLG_1818	Sputum	USA	02/28/2009	NEQE01	KL2

AS012522	Lung	USA	03/14/2016	VKRC01	KL2
AS012524	Lung	USA	04/26/2016	VKRA01	KL2
AS012543	Lung	USA	09/15/2015	VLXR01	KL2
AYP-A2	Wound	Australia	10/11/2013	CP024124	KL2
BIDMC_56	Urine	USA	07/01/2013	JMUH01	KL2
BJ2	Sputum	China	07/01/2012	JPLG01	KL2
BJ6	Sputum	China	07/01/2012	JPLK01	KL2
BJ7	Sputum	China	07/01/2012	JPLL01	KL2
BP629	Blood	India	07/01/2019	JAAEFN01	KL2
BP723	Blood	India	07/01/2019	JAAGSD01	KL2
CAb24_1	Bronchoalveolar Lavage Fluid	China	02/20/2018	VCNR01	KL7
CAb24_2	Bronchoalveolar Lavage Fluid	China	02/20/2018	VCNQ01	KL7
CAb24_3	Sputum	China	02/28/2018	VCNT01	KL7
CBA7	Sputum	South Korea	10/01/2013	CP020586	KL2
CCF18	Tissue/Wound/Sacral	Unknown	07/15/2001	LZAL01	KL2
CCF21	Blood	Unknown	01/19/2014	LZAO01	KL2
CCF25	Wound Swab	Unknown	12/15/2010	LZAS01	KL2
CCF38	Blood	Unknown	02/12/2011	LYZR01	KL124
CCF42	Blood	Unknown	01/13/2007	LYZU01	KL2
CCF49	Blood	Unknown	07/15/2012	LZBE01	KL2
CCF62	Leg	Unknown	10/30/2014	LZBP01	KL2
CCF73	Blood	Unknown	01/13/2002	LZBZ01	KL2
CCF9	Tracheal Aspirate	Unknown	08/28/2014	LZAC01	KL124
CFSAN059604	Urine	Pakistan	07/01/2004	SSMO01	KL2
CI77	Superficial Wound	Iraq	07/01/2005	AVOC01	KL2
CU060707	Blood	USA	06/07/2007	LXWY01	KL2
H350	Blood	Mexico	07/01/2015	SGSF01	KL2
HJ01	Kidney Preservation Fluid	China	07/01/2013	LYTI01	KL2
HJ07	Kidney Preservation Fluid	China	07/01/2013	MACT01	KL7
HJ08	Kidney Preservation Fluid	China	07/01/2013	MACU01	KL7
HJ13	Kidney Preservation Fluid	China	07/01/2014	MACY01	KL2
HRAB_85	Sputum	China	09/01/2014	CP018143	KL2
ISAB51	Blood	China	07/01/2010	LFHC01	KL2
J133	Unknown	Japan	07/01/2010	LAKQ01	KL2
J65	Unknown	Japan	07/01/2010	LAKO01	KL2
KAB08	Wound	South Korea	09/10/2015	CP017656	KL9
LY4	Unknown	China	03/09/2012	JDSY01	KL2
LY5	Unknown	China	07/01/2012	JDSZ01	KL2
LY9	Unknown	China	04/01/2012	JDTD01	KL2
MDR_CQA	Unknown	China	04/01/2017	SNVT01	KL7
MDR_CQE	Unknown	China	05/01/2017	SNVP01	KL2
MDR_CQF	Unknown	China	04/01/2017	SNVO01	KL7
MDR_CQH	Unknown	China	04/01/2017	SNVM01	KL2
MDR_CQI	Unknown	China	04/01/2017	SNVL01	KL2
MDRAB55	Blood	China	07/01/2009	LFHK01	KL2
MDRAB7	Blood	China	07/01/2010	LFHM01	KL2
MH3	Unknown	China	07/01/2011	QPHQ01	KL2
MRSN16874	Blood	USA	07/01/2011	LNBD01	KL2
MRSN7431	Respiratory	USA	07/01/2005	VHEC01	KL2
N13_03449	Unknown	Canada	07/01/2013	CP043417	KL9
Naval_113	Human Wound	USA	07/01/2002	AMZU01	KL2
NIPH_2061	I.V. Cannula	Czech Republic	07/01/2003	APOW01	KL2
NSAb24_1	Nasopharynx	China	02/28/2018	VCNU01	KL7
NSAb24_2	Nasopharynx	China	02/20/2018	VCNS01	KL7
O1_4	Sputum	China	01/14/2018	SDBE01	KL2
O1_8	Sputum	China	01/28/2018	SDBI01	KL2
O2_4	Sputum	China	08/08/2018	SEIK01	KL2
OIFC189	Unknown	USA	07/01/2003	AFDM01	KL2
ORAB01	Bodily Fluid	USA	06/14/2012	CP015483	KL2
PB364	Unknown	USA	07/01/2016	CP040425	KL2
PU_2016_41	Blood	Greece	07/01/2016	WIHS01	KL2
R1	Bal	USA	01/24/2007	PUCB01	KL2
R10	Blood	USA	03/11/2014	PUCT01	KL2
R11	Sputum	USA	07/11/2015	PUCV01	KL2
R12	Mediastinal Swab	USA	01/19/2007	PUCX01	KL2
R13	Sputum	USA	07/02/2015	PUCZ01	KL2
R15	Bal	USA	06/04/2008	PUDD01	KL2

R16	Sputum	USA	08/21/2009	PUDF01	KL2
R17	SPUT	USA	05/22/2010	PUDH01	KL2
R2	Bal	USA	02/16/2011	PUCD01	KL2
R4	Bronch Wash	USA	07/21/2011	PUCH01	KL2
R5	Bal	USA	06/30/2012	PUCJ01	KL2
R7	Bronch Wash	USA	06/16/2010	PUCN01	KL2
R9	Bal	USA	01/25/2011	PUCR01	KL2
RCS6	Urine	France	07/18/2018	SAXN01	KL2
S10	Blood	USA	02/03/2014	PUCS01	KL2
S11	Bal	USA	06/29/2015	PUCU01	KL2
S12	Mediastinal Fluid	USA	01/12/2007	PUCW01	KL2
S13	Bronch Wash	USA	07/07/2015	PUCY01	KL2
S14	Bronch Wash	USA	09/16/2015	PUDA01	KL2
S15	Bal	USA	02/26/2008	PUDC01	KL2
S16	Bal	USA	08/17/2009	PUDE01	KL2
S17	SPUT	USA	05/02/2010	PUDG01	KL2
S2	Bal	USA	02/05/2011	PUCC01	KL2
S20	Sputum	USA	10/23/2015	PUDM01	KL2
S3	Bal	USA	01/13/2011	PUCE01	KL2
S4	Bal	USA	07/10/2011	PUCG01	KL2
S5	Bal	USA	05/27/2012	PUCI01	KL2
S7	Bal	USA	06/01/2010	PUCM01	KL2
S8	Trach Aspirate	USA	12/20/2010	PUCO01	KL2
S9	Bal	USA	12/14/2010	PUCQ01	KL2
SGH0825	Tracheal Aspirate	Singapore	07/01/2008	PYEI01	KL2
SI_2017_63	Bronchial Secretions	Greece	07/01/2017	WIHA01	KL2
SI_2017_66	Pleural Fluid	Greece	07/01/2017	WIGY01	KL2
SIUA14	Patient	Thailand	06/11/2016	NIUL01	KL2
SPU6	Sputum	China	07/01/2009	LFHN01	KL2
SSMA8	Clean Voided Urine	South Korea	10/13/2020	NBNJ01	KL2
strainS1	Bal	USA	01/09/2007	CP026943	KL2
T7	Unknown	Thailand	01/10/2020	JRQT01	KL2
TCM331	Sputum	China	11/22/2012	LGHQ01	KL2
TG15428	Sputum	Unknown	11/08/2010	RFAZ01	KL2
TG15458	Environmental	USA	11/08/2010	RFBT01	KL2
TG15461	Wound	Unknown	11/08/2010	RFBJ01	KL2
TG15470	Environmental	Unknown	11/08/2010	RFDW01	KL2
TG16732	Unknown	USA	11/03/2010	RFBI01	KL2
TG16750	Unknown	USA	11/03/2010	RFCZ01	KL2
TG22180	Wound	USA	07/19/2011	RFCK01	KL2
TG22206	Sputum	USA	07/19/2011	RFCT01	KL2
TG22653	Bal	USA	10/15/2011	CP039518	KL2
TG27303	Sputum	USA	12/22/2011	RFDP01	KL2
TG29391	Blood	USA	03/06/2012	RFDA01	KL2
TG31971	Urine	USA	03/12/2012	RFCF01	KL2
TG31982	Wound	USA	03/12/2012	RFBH01	KL2
TG40843	Blood	USA	07/06/2012	RFBP01	KL2
TG40983	Trach Asp	USA	07/06/2012	RFCV01	KL2
TG60017	Trach Asp	USA	04/04/2013	RFDD01	KL2
TG60019	Trach Asp	USA	04/11/2013	RFCJ01	KL2
TG60536	Sputum	USA	04/18/2013	RFDV01	KL2
TG80268	Blood	USA	11/20/2015	RFBX01	KL2
TG89536	URT	USA	11/20/2015	RFDK01	KL2
TG89540	Blood	USA	11/20/2015	RFCQ01	KL2
TG91920	Blood	USA	11/20/2015	RFDQ01	KL2
TR_2016_35	Blood	Greece	07/01/2016	WIHX01	KL2
UH0207	Sputum	USA	11/07/2009	AYGS01	KL2
UH10007	Sputum	USA	12/21/2007	AYGO01	KL2
UH1007	Wound Abscess	USA	07/01/2007	AYGP01	KL2
UH10107	Bronchial Wash	USA	12/22/2007	AYGN01	KL2
UH11608	Urine	USA	08/08/2001	AYGL01	KL2
UH12308	Catheter	USA	01/17/2008	AYGJ01	KL2
UH12408	Sputum	USA	01/18/2008	AYGI01	KL2
UH12808	Trach Tube	USA	01/23/2008	AYGH01	KL2
UH13908	Bronchial Wash	USA	02/26/2008	AYGG01	KL2
UH14508	Blood	USA	10/08/2003	AYGF01	KL2
UH15208	Sputum	USA	03/18/2008	AYGE01	KL2

UH16008	Wound	USA	03/25/2008	AYGD01	KL2
UH16108	Sputum	USA	03/25/2008	AYGC01	KL2
UH17_151	Bodily Fluid	USA	03/18/2008	JWXB03	KL2
UH18608	Sputum	USA	04/23/2008	AYGA01	KL2
UH19908	Sputum	USA	12/08/2005	AYFY01	KL2
UH20108	Catheter	USA	05/14/2008	AYFX01	KL2
UH2107	Sputum	USA	05/31/2007	AYFW01	KL2
UH225_433	Bodily Fluid	USA	10/22/2009	JWVA03	KL2
UH2307	Trach Tube	USA	06/14/2007	AYFU01	KL2
UH3807	Sputum	USA	09/07/2010	AYFR01	KL2
UH514_289	Sputum	USA	01/08/2009	JZJG02	KL2
UH5307	Sputum	USA	12/07/2010	AYFO01	KL2
UH5707	Wound	USA	11/07/2010	AYFN01	KL2
UH6107	Sputum	USA	10/13/2007	AYFM01	KL2
UH6207	Bronchial Wash	USA	10/22/2007	AYFL01	KL2
UH66_253	Fluid	USA	07/16/2008	JZIZ02	KL2
UH66_271	Wound	USA	08/15/2008	JZIX02	KL2
UH7807	Sputum	USA	07/07/2011	AYFF01	KL2
UH8107	Bronchial Wash	USA	11/21/2007	AYFD01	KL2
UH8407	Blood	USA	11/25/2007	AYFC01	KL2
UH8707	Biopsy	USA	11/25/2007	AYFB01	KL2
UH8807	Sputum	USA	02/07/2012	AYFA01	KL2
UH8907	Blood	USA	02/07/2012	AYEZ01	KL2
UH9007	Blood	USA	03/07/2012	AYEY01	KL2
UH9707	Catheter	USA	12/18/2007	AYEX01	KL2
UH9907_1	Sputum	USA	12/20/2007	AYEW01	KL2
UH9907_2	Sputum	USA	07/01/2007	AYOH01	KL2
VB723	Blood	India	07/01/2019	CP050390	KL2
WM99c	Unknown	Australia	07/01/1999	CP031743	KL2
XH386_1	Lower Respiratory Tract	China	05/29/2014	CP010779	KL2
XH386_2	Unknown	China	07/01/2017	CP021326	KL2
XH506	Sputum	China	06/20/2014	LYLM01	KL7
XH507	Sputum	China	09/14/2014	LYLL01	KL7
XH546	Sputum	China	08/17/2014	LYLB01	KL7
XH549	Sputum	China	09/15/2014	LYKY01	KL2
XH550	Sputum	China	11/05/2014	LYKX01	KL2
XH667	Sputum	China	02/12/2015	LYJW01	KL2
XH671	Sputum	China	01/26/2015	LYJS01	KL2
XH672	Sputum	China	03/10/2015	LYJR01	KL2
XH684	Sputum	China	08/24/2012	LYJF01	KL2
XH686	Sputum	China	03/14/2014	LYJD01	KL2
XH698	Sputum	China	06/18/2013	LYIV01	KL2
XH706	Sputum	China	10/22/2014	LYIP01	KL2
XH727	Sputum	China	11/08/2013	LYHY01	KL7
XH728	Sputum	China	02/06/2014	LYHX01	KL7
XH733	Sputum	China	06/02/2013	LYHS01	KL2
XH747	Sputum	China	11/30/2013	LYHF01	KL2
XH748	Sputum	China	06/28/2013	LYHE01	KL2
XH780	Sputum	China	07/04/2013	LYFE01	KL2
XH804	Sputum	China	01/21/2014	LYFU01	KL2
XH823	Sputum	China	02/20/2013	LYFO01	KL2
XH835	Sputum	China	03/06/2014	LYFI01	KL2
XH839	Sputum	China	04/12/2012	LYEY01	KL2
YMC2011_2_C582	Swab Or Tube Drainage Buttock	South Korea	02/15/2011	MKHK01	KL2
YMC2012_7_R3167	Sputum	South Korea	07/15/2012	MKHJ01	KL2
ZS3	Unknown	China	06/01/2009	CP021496	KL2

Table S2. 76 ST208 *A. baumannii* plasmids involved in this study

Strain	Plasmid	Length	Blast Results	Accession	% Identity	Rep group
5388	p5388_2	110966	Acinetobacter baumannii MDR-TJ plasmid pABTJ2, complete sequence	CP004359.1	99	GR24
5388	p5388_1	77533	Acinetobacter baumannii MDR-TJ plasmid pABTJ1, complete sequence	CP003501.1	99	GR25
5626	p5626	110967	Acinetobacter baumannii MDR-TJ plasmid pABTJ2, complete sequence	CP004359.1	99	GR24
5634	p5634	110970	Acinetobacter baumannii MDR-TJ plasmid pABTJ2, complete sequence	CP004359.1	99	GR24
5651	p5651	72248	Acinetobacter baumannii TYTH-1 plasmid pAB_CC, complete sequence	KF889012.1	99	GR6
5653	p5653_2	11194	Acinetobacter baumannii strain VB23193 plasmid unnamed1, complete sequence	CP035673.1	99	GR8
5653	p5653_1	110967	Acinetobacter baumannii MDR-TJ plasmid pABTJ2, complete sequence	CP004359.1	99	GR24
5656	p5656	11194	Acinetobacter baumannii strain VB23193 plasmid unnamed1, complete sequence	CP035673.1	99	GR8
5662	p5662	77530	Acinetobacter baumannii MDR-TJ plasmid pABTJ1, complete sequence	CP003501.1	99	GR25
5663	p5663	71044	Acinetobacter baumannii strain 7835 plasmid pAba7835b, complete sequence	CP033245.1	99	GR6
5664	p5664	71044	Acinetobacter baumannii strain 7835 plasmid pAba7835b, complete sequence	CP033245.1	99	GR6
5665	p5665_3	11205	Acinetobacter baumannii strain VB35575 plasmid unnamed1, complete sequence	CP040088.1	99	GR8
5665	p5665_2	110967	Acinetobacter baumannii MDR-TJ plasmid pABTJ2, complete sequence	CP004359.1	99	GR24
5665	p5665_1	77530	Acinetobacter baumannii MDR-TJ plasmid pABTJ1, complete sequence	CP003501.1	99	GR25
5666	p5666_3	110967	Acinetobacter baumannii MDR-TJ plasmid pABTJ2, complete sequence	CP004359.1	99	GR24
5666	p5666_1	11194	Acinetobacter baumannii strain VB35575 plasmid unnamed1, complete sequence	CP040088.1	99	GR8
5666	p5666_2	77530	Acinetobacter baumannii MDR-TJ plasmid pABTJ1, complete sequence	CP003501.1	99	GR25
5669	p5669	112152	Acinetobacter baumannii MDR-TJ plasmid pABTJ2, complete sequence	CP004359.1	99	GR24
5670	p5670	112156	Acinetobacter baumannii MDR-TJ plasmid pABTJ2, complete sequence	CP004359.1	99	GR24
5671	p5671_1	110967	Acinetobacter baumannii MDR-TJ plasmid pABTJ2, complete sequence	CP004359.1	99	GR24
5671	p5671_2	71278	Acinetobacter baumannii strain MDR-CQ plasmid pMDR-CQ, complete sequence	CP019115.1	99	GR6
5672	p5672	11196	Acinetobacter baumannii strain VB35575 plasmid unnamed1, complete sequence	CP040088.1	99	GR8
5678	p5678_1	110998	Acinetobacter baumannii MDR-TJ plasmid pABTJ2, complete sequence	CP004359.1	99	GR24
5678	p5678_2	77537	Acinetobacter baumannii MDR-TJ plasmid pABTJ1, complete sequence	CP003501.1	99	GR25
5678	p5678_3	11194	Acinetobacter baumannii strain VB23193 plasmid unnamed1, complete sequence	CP035673.1	99	GR8
5679	p5679	73209	Acinetobacter baumannii TYTH-1 plasmid pAB_CC, complete sequence	KF889012.1	99	GR6
5683	p5683_2	110968	Acinetobacter baumannii MDR-TJ plasmid pABTJ2, complete sequence	CP004359.1	99	GR24
5683	p5683_1	77540	Acinetobacter baumannii MDR-TJ plasmid pABTJ1, complete sequence	CP003501.1	99	GR25
5689	p5689_1	110967	Acinetobacter baumannii MDR-TJ plasmid pABTJ2, complete sequence	CP004359.1	99	GR24
5689	p5689_2	71479	Acinetobacter baumannii MDR-TJ plasmid pABTJ1, complete sequence	CP003501.1	99	GR25
5729	p5729	77542	Acinetobacter baumannii MDR-TJ plasmid pABTJ1, complete sequence	CP003501.1	99	GR25
5732	p5732	110968	Acinetobacter baumannii MDR-TJ plasmid pABTJ2, complete sequence	CP004359.1	99	GR24
5734	p5734_2	110965	Acinetobacter baumannii MDR-TJ plasmid pABTJ2, complete sequence	CP004359.1	99	GR24
5734	p5734_1	77534	Acinetobacter baumannii MDR-TJ plasmid pABTJ1, complete sequence	CP003501.1	99	GR25
5736	p5736	112157	Acinetobacter baumannii MDR-TJ plasmid pABTJ2, complete sequence	CP004359.1	99	GR24
5740	p5740	112155	Acinetobacter baumannii MDR-TJ plasmid pABTJ2, complete sequence	CP004359.1	99	GR24
5741	p5741_2	70793	Acinetobacter baumannii TYTH-1 plasmid pAB_CC, complete sequence	KF889012.1	99	GR6
5741	p5741_1	21174	Acinetobacter baumannii strain VB31459 plasmid unnamed2, complete sequence	CP035932.1	99	NA
5745	p5745_2	77530	Acinetobacter baumannii MDR-TJ plasmid pABTJ1, complete sequence	CP003501.1	99	GR25
5745	p5745_1	44752	Acinetobacter baumannii strain VB23193 plasmid unnamed2, complete sequence	CP035674.1	99	GR8
5759	p5759_2	77531	Acinetobacter baumannii MDR-TJ plasmid pABTJ1, complete sequence	CP003501.1	99	GR25
5759	p5759_1	110967	Acinetobacter baumannii MDR-TJ plasmid pABTJ2, complete sequence	CP004359.1	99	GR24
5759	p5759_3	33550	Acinetobacter baumannii strain VB23193 plasmid unnamed2, complete sequence	CP035674.1	99	GR8
5760	p5760_2	110967	Acinetobacter baumannii MDR-TJ plasmid pABTJ2, complete sequence	CP004359.1	99	GR24
5760	p5760_1	77531	Acinetobacter baumannii MDR-TJ plasmid pABTJ1, complete sequence	CP003501.1	99	GR25
5761	p5761	71277	Acinetobacter baumannii strain MDR-CQ plasmid pMDR-CQ, complete sequence	CP019115.1	99	GR6
5767	p5767	70287	Acinetobacter baumannii MDR-TJ plasmid pABTJ1, complete sequence	CP003501.1	99	GR25
5768	p5768	110966	Acinetobacter baumannii MDR-TJ plasmid pABTJ2, complete sequence	CP004359.1	99	GR24
5769	p5769	110970	Acinetobacter baumannii MDR-TJ plasmid pABTJ2, complete sequence	CP004359.1	99	GR24
5771	p5771	112156	Acinetobacter baumannii MDR-TJ plasmid pABTJ2, complete sequence	CP004359.1	99	GR24
5773	p5773_1	22354	Acinetobacter baumannii strain VB23193 plasmid unnamed2, complete sequence	CP035674.1	99	GR8
5773	p5773_2	78842	Acinetobacter baumannii MDR-TJ plasmid pABTJ1, complete sequence	CP003501.1	99	GR25
5836	p5836	78021	Acinetobacter baumannii TYTH-1 plasmid pAB_CC, complete sequence	KF889012.1	99	GR6
5839	p5839	71277	Acinetobacter baumannii strain KAB05 plasmid, complete sequence	CP017651.1	99	GR6
5840	p5840	73209	Acinetobacter baumannii TYTH-1 plasmid pAB_CC, complete sequence	KF889012.1	99	GR6
5846	p5846	110968	Acinetobacter baumannii MDR-TJ plasmid pABTJ2, complete sequence	CP004359.1	99	GR24
5847	p5847	110973	Acinetobacter baumannii MDR-TJ plasmid pABTJ2, complete sequence	CP004359.1	99	GR24
5955	p5955	22367	Acinetobacter baumannii strain VB23193 plasmid unnamed1, complete sequence	CP035673.1	99	GR8
7847	pAba7847a	13478	NA	CP023032	NA	GR26
7847	pAba7847b	80546	NA	CP023033	NA	GR6
ABAY04001	pABAY04001_1A	110967	NA	NZ_MK386680	NA	GR24
AF-673	PAF-673	110964	NA	NZ_CP018257	NA	GR24
AYP-A2	pAYP-A2	110967	NA	NZ_CP024125	NA	GR24
CBA7	pCBA7_1	111999	NA	NZ_CP020585	NA	GR24
HRAB-85	HRAB-85 plasmid	77513	NA	NZ_CP018144	NA	GR25
KAB08	KAB08 plasmi	101406	NA	NZ_CP017657	NA	GR6
OIFC189	pOIFC189-111	110967	NA	NZ_AFDM01000010	NA	GR24
ORAB01	pORAB01-3	15198	NA	NZ_CP015486	NA	GR2
ORAB01	pORAB01-1	110965	NA	NZ_CP015484	NA	GR24
ORAB01	pORAB01-2	24022	NA	NZ_CP015485	NA	NA
strain S1	pAbS1_02	111068	NA	NZ_CP026945	NA	GR24
UH2107	pABUH4-111	111007	NA	NZ_AYFW01000101	NA	GR24
UH9707	pABUH6a-8.8	8763	NA	NZ_AYEX01000118	NA	GR2
UH9907	pABUH1-74	74089	NA	NZ_AYOH01000010	NA	GR6
WM99c	pWM99c-2	110967	NA	CP031744.1	NA	GR24
XH386	pAB386	112157	NA	NZ_CP010780	NA	GR24
